# Evaluating nephrotoxicity reduction in a novel polymyxin B formulation: insights from a 3D kidney-on-a-chip model

**DOI:** 10.1128/aac.00219-24

**Published:** 2024-09-03

**Authors:** Anurag Payasi, Manoj Kumar Yadav, Saransh Chaudhary, Anmol Aggarwal

**Affiliations:** 1 Department of Cell Culture, Venus Medicine Research Centre, Baddi, Himachal Pradesh, India; 2 Venus Medicine Research Centre, Baddi, Himachal Pradesh, India; 3 Department of Pipeline Strategy, Venus Medicine Research Centre, Baddi, Himachal Pradesh, India; University of Pittsburgh, Pittsburgh, Pennsylvania, USA

**Keywords:** polymyxin B, microphysiological system, kidney-on-a-chip, nephrotoxicity, proximal tubule, VRP-034

## Abstract

This study aimed to assess the nephrotoxicity associated with VRP-034 (novel formulation of polymyxin B [PMB]) compared to marketed PMB in a three-dimensional (3D) kidney-on-a-chip model. To model the human kidney proximal tubule for analysis, tubular structures were established using 23 triple-channel chips seeded with RPTEC/hTERT1 cells. These cells were exposed to VRP-034 or PMB at seven concentrations (1–200 µM) over 12, 24, and 48 h. A suite of novel kidney injury biomarkers, cell health, and inflammatory markers were quantitatively assessed in the effluent. Additionally, caspase and cytochrome C levels were measured, and cell viability was evaluated using calcein AM and ethidium homodimer-1 (EthD-1). Exposure to marketed PMB resulted in significantly elevated levels (*P* < 0.05) of four key biomarkers (KIM-1, cystatin C, clusterin, and OPN) compared to VRP-034, particularly at clinically relevant concentrations of ≥10 µM. At 25 µM, all biomarkers demonstrated a significant increase (*P* < 0.05) with marketed PMB exposure compared to VRP-034. Inflammatory markers (interleukin-6 and interleukin-8) increased significantly (*P* < 0.05) with marketed PMB at concentrations of ≥5 µM, relative to VRP-034. VRP-034 displayed superior cell health outcomes, exhibiting lower lactate dehydrogenase release, while ATP levels remained comparable. Morphological analysis revealed that marketed PMB induced more severe damage, disrupting tubular integrity. Both treatments activated cytochrome C, caspase-3, caspase-8, caspase-9, and caspase-12 in a concentration-dependent manner; however, caspase activation was significantly reduced (*P* < 0.05) with VRP-034. This study demonstrates that VRP-034 significantly reduces nephrotoxicity compared to marketed PMB within a 3D microphysiological system, suggesting its potential to enable the use of full therapeutic doses of PMB with an improved safety profile, addressing the need for less nephrotoxic polymyxin antibiotics.

## INTRODUCTION

The escalating threat of antimicrobial resistance poses a formidable challenge to global public health, jeopardizing our ability to effectively combat infectious diseases (1–3). Among the dwindling arsenal of effective antibiotics, polymyxins have garnered renewed attention as a crucial last-line defense against multidrug-resistant (MDR) pathogens (4–6). However, the clinical utility of polymyxins is hampered by their potential to induce nephrotoxicity, resulting in acute kidney injury (AKI). Clinical studies have reported that intravenous administration of polymyxins causes AKI in as many as 60% of patients (7, 8). Although polymyxins remain essential in combating MDR infections, their use is declining worldwide and particularly in the United States, as healthcare policies increasingly aim to reduce the usage of toxic drugs (9). Thus, it is crucial to understand and mitigate polymyxin-induced nephrotoxicity to preserve these vital antibiotics and safeguard patient well-being.

While the exact mechanism of nephrotoxicity associated with polymyxins remains elusive, earlier research has suggested that following glomerular filtration, polymyxins accumulate in proximal tubular cells through receptor-mediated endocytosis, primarily involving megalin/cubulin receptors (10, 11) and the PEPT2 transporter (12). This accumulation triggers various pathways, including the death receptor pathway, mitochondrial pathway, and endoplasmic reticulum pathway, which are primarily mediated by caspases (13–17). Activation of these pathways results in the excessive generation of pathological reactive oxygen species (ROS), potentially leading to an imbalance in the oxidant/antioxidant system; structural and functional alterations in cellular components such as proteins, lipids, and DNA; damage to lipid membranes; inflammation; tissue damage; mitochondrial dysfunction; apoptosis; and ultimately, cell death (15, 18, 19).

In the realm of polymyxin therapeutics, the significant nephrotoxicity associated with agents such as colistin (also known as polymyxin E) and polymyxin B (PMB) necessitates the exploration of formulations that mitigate this deleterious effect while preserving antimicrobial potency. Historically, modifications to the molecular structure of PMB have been pursued to attenuate renal toxicity; however, these alterations have often led to compromises in either the drug’s efficacy or its pharmacokinetic profile (20, 21). In deviation from these conventional methodologies, VRP-034 embodies an innovative approach through the creation of a supramolecular cationic (SMC) formulation that integrates PMB, L-arginine, and low-molecular-weight dextran. This formulation distinguishes itself by leveraging electrostatic interactions within a meticulously defined charge-to-molecular weight ratio, aiming to maintain the intrinsic antimicrobial efficacy of PMB. It is engineered to diminish the oxidative stress associated with PMB accumulation and to enhance crucial microcirculatory functions essential for maintaining kidney health. The proposed mechanism of action for SMC involves targeting oxidative stress through the inhibition of pathways that lead to cellular damage. Additionally, molecular simulations and two-dimensional (2D) ROESY NMR data (data not shown) suggest that L-arginine-PMB interactions within the SMC complex may disrupt PMB’s binding to receptors involved in renal uptake, potentially reducing PMB’s accumulation in renal cells. Although direct experimental validation of this hypothesis is pending, VRP-034 introduces a novel strategy to address polymyxin-induced nephrotoxicity, setting a new direction for research into its therapeutic capabilities without pre-emptively asserting its safety, which is rigorously assessed in the present study.

Examining the formulation’s ability to mitigate renal toxicity is challenging. Ethical constraints and practical limitations hinder the direct study of kidney toxicity mechanisms directly in humans, and conventional preclinical models, like animal studies, encounter issues related to species differences and translatability of findings to human outcomes. In recent years, there has been a growing interest in three-dimensional (3D) microphysiological systems (MPSs) like organ-on-a-chip models, as a solution to the limitations of 2D *in vitro* cell cultures and animal models. These advanced systems, particularly the kdney-on-a-chip model, are designed to predict nephrotoxicity in drug development processes by more accurately emulating human physiological conditions *in vitro*. This approach aims to bridge the existing gap between *in vitro* and *in vivo* testing, facilitating the early detection of human toxicity. Cells cultured in 3D kidney-on-a-chip model more accurately replicate *in vivo* conditions, encompassing cell morphology, structural properties, mechanical behavior, transport mechanisms, absorptive capacities, protein and biomarker expressions, gene profiles, and the physiological characteristics of human kidney proximal tubules (22–27).

**Fig 1:**
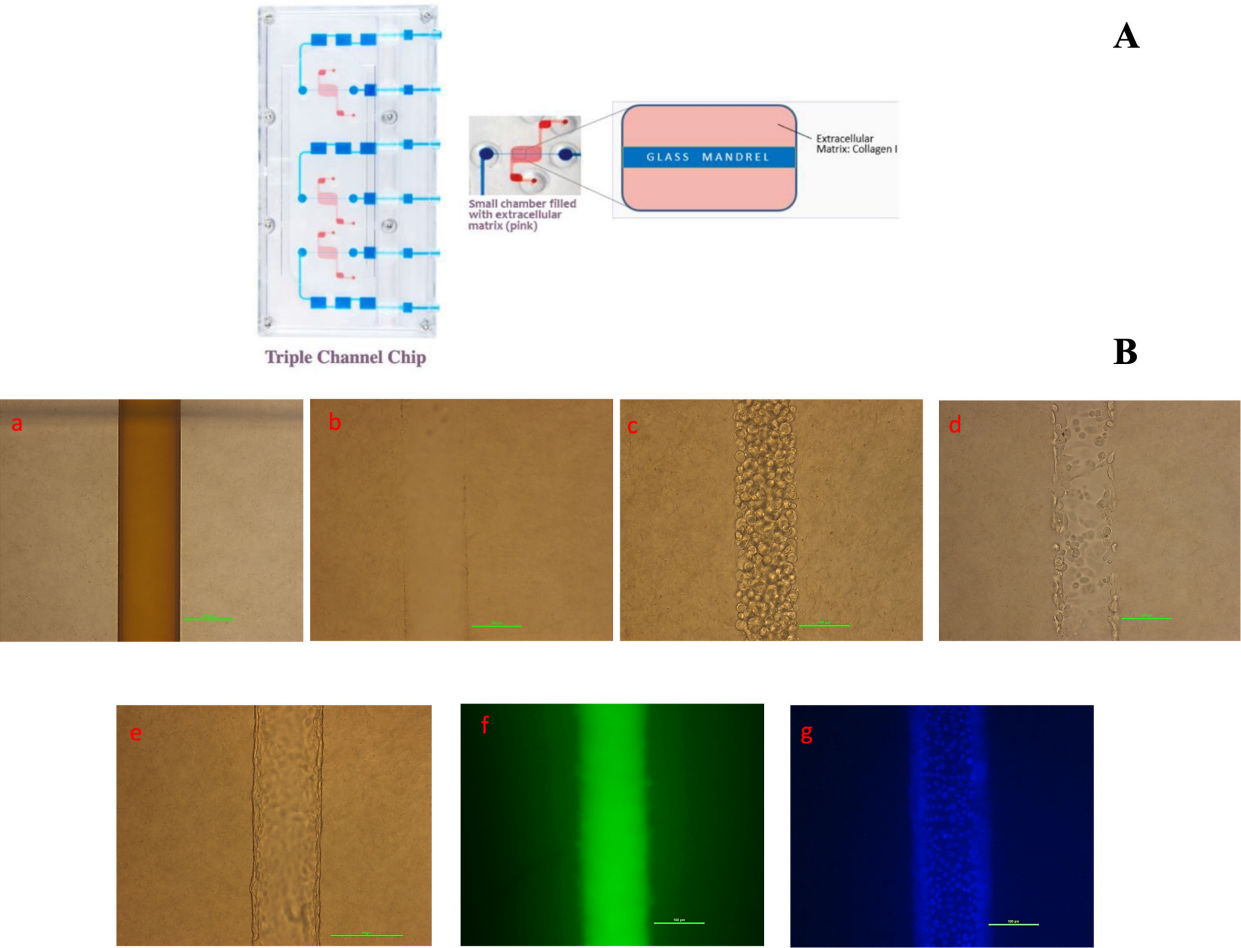
Image of the chip and the tubule formation inside the channel of chip. (A) Image of the triple-channel chips (Source: Nortis Bio, USA). (B) Phase-contrast image showing the step-wise establishment of tubules of RPTEC/TERT-1 cells in the channels of chip/MPS: (a) channel with glass mandrel; (b) empty channel after removing the glass mandrel; (c) channel seeded with RPTEC/hTERT1 cells; (d) channel after removing un-attached cells after overnight attachment; (e) viability of RPTEC/hTERT1 cells in the MPS/chip at day 9 of post-seeding: RPTEC/hTERT1 cells showing confluent tube/tubule are formed in the channel; (f) barrier integrity assay: a fluorescent dye [150-kDa fluorescein isothiocyanate (FITC)-dextran] was inserted in the channel comprising the tube. The integrity of the tube barrier is quantified by observing the dye that is leaking out of the tube into the adjacent channel. (g) Hoechst (indicator of total cells) fluorescent image of nuclei of cells forming tubules was also analyzed: the morphology of the cell tubules is seen to be fully intact. Scale bar: 100 µm (×20 magnification).

In the current investigation, we developed a 3D MPS model using renal proximal tubule epithelial cells (RPTECs/hTERT1). This model was constructed utilizing the triple-channel chips and the microfluidic platform provided by Nortis Bio (Seattle, USA), as depicted in Fig. 1. The system facilitated a comparative analysis of the nephrotoxic potential between VRP-034 and a commercially available formulation of PMB, hereafter referred to as marketed PMB. The evaluation employed a range of markers to gauge kidney injury, including the release of novel biomarkers such as kidney injury molecule-1 (KIM-1), cystatin-C, clusterin, N-acetyl-β-D-glucosaminidase (NAG), neutrophil gelatinase-associated lipocalin (NGAL), and osteopontin (OPN). Additionally, the study assessed the release of inflammatory biomarkers, including interleukin-6 (IL-6) and interleukin-8 (IL-8), evaluated cell health via lactate dehydrogenase (LDH) and adenosine triphosphate (ATP) measurements, analyzed toxicity pathways, monitored cellular stress, and assessed cell viability and mortality.

## RESULTS

### Establishment of RPTEC/TERT-1 cells tubules

The Nortis Bio triple single-channel (TSC) chip used in this study, which incorporates an extracellular matrix compartment (depicted in red) housing three channels (illustrated within a gray box), is showcased in Fig. 1A. The sequential development of RPTEC/hTERT1 cell tubules within the MPS chip is detailed in Fig. 1B. After seeding cells in the channels, RPTEC/hTERT1 cells successfully adhered to the substrate and proliferated, culminating in the formation of structures akin to tubules that mimic the proximal segments of human renal tubules in their physical dimensions. The integrity of these tubule-like formations was verified through the application of FITC-dextran staining, which confirmed the absence of leakage. Additionally, the tubule-forming cells demonstrated a high rate of viability, exceeding 95%, as evidenced by the intense blue fluorescence resulting from Hoechst staining, which marks live cells.

### Cell health biomarkers

The ATP and LDH levels from the untreated controls served as baseline metrics for assessing the effect of the compounds. The findings revealed that exposure to both compounds led to a reduction in ATP levels in a concentration-dependent manner across all time points, with no notable distinction between the effects of the two compounds. Conversely, the release of intracellular LDH was significantly elevated (*P* ≤ 0.05) following administration of 5, 10, and 25 µM of marketed PMB in comparison to VRP-034, indicating a marked difference in cytotoxicity induced by the two substances.

### Kidney injury biomarkers

**Fig 2:**
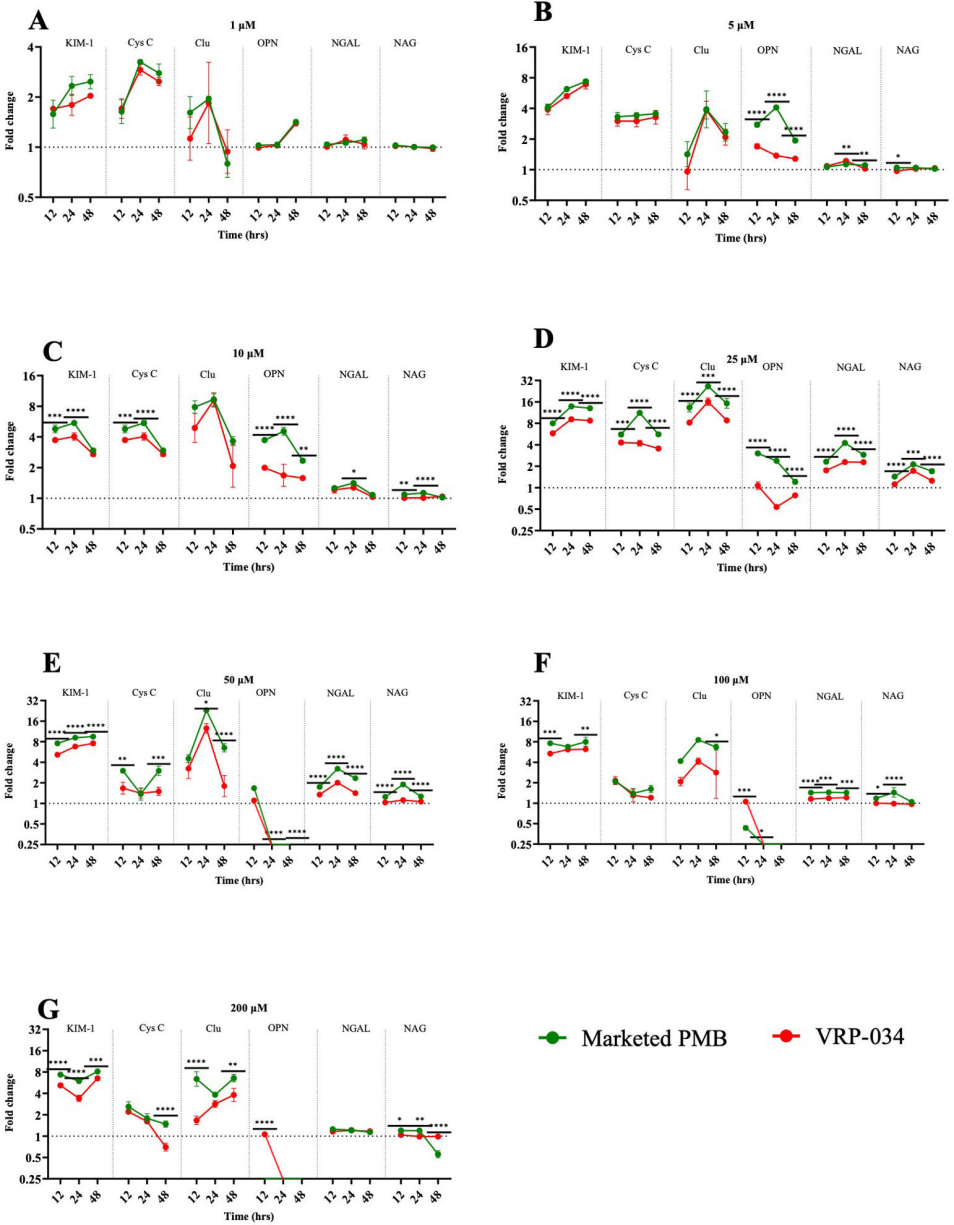
Biomarker levels after 12-, 24-, and 48-h exposures with marketed PMB and VRP-034. (A–G) Levels of KIM-1, cystatin-C, clusterin, NAG, NGAL, and osteopontin in the effluent of human RPTEC/hTERT1 cells after 12, 24, and 48 h of exposure with different concentrations of marketed PMB and VRP-034. Data presented as the geo-mean (±SD) of the fold change from control. *P* values are calculated for the log-transformed fold-change values between the two drugs at a particular time point. Significance is denoted by *P < 0.05, **P < 0.01, ***P < 0.001, ****P < 0.0001.

A noticeable increase in the levels of four (out of six) novel biomarkers (KIM-1, cystatin C, clusterin, and OPN) was observed following exposure to both drugs, particularly at and above clinically relevant concentrations of 10 µM (Fig. 2). However, the rise in biomarker levels was notably less pronounced with VRP-034 than with marketed PMB. Specifically, at a 25-µM concentration, a statistically significant elevation (*P* < 0.001) in all six biomarkers was detected in the group exposed to marketed PMB compared to the VRP-034 group across all measured time points. The peak in biomarker release was generally observed at the 24-h mark.

### Inflammatory markers

**Fig 3:**
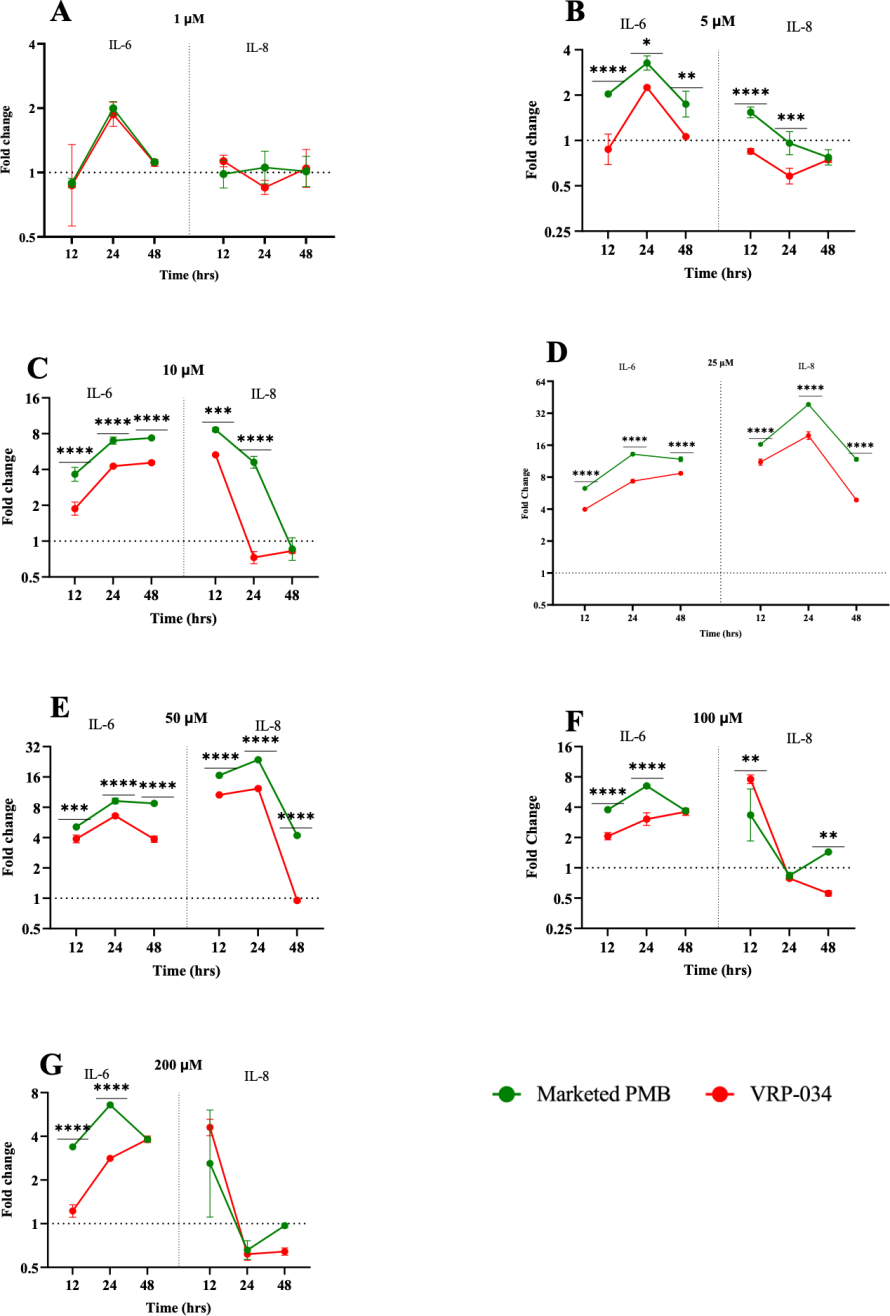
Inflammatory markers levels after 12-, 24-, and 48-h exposures with marketed PMB and VRP-034. (A–G) Inflammatory marker (IL-6 and IL-8) levels after 12, 24, and 48 h of exposure with different concentrations of marketed PMB and VRP-034. Data presented as the geo-mean (±SD) of the fold change from control. *P* values are calculated for the log-transformed fold-change values between the two drugs at a particular time point. Significance is denoted by *P < 0.05, **P < 0.01, ***P < 0.001, ****P < 0.0001.

A substantial increase (*P* ≤ 0.05) in the levels of IL-6 and IL-8 was documented following exposure to marketed PMB, especially at concentrations of 5 µM and above, at all assessed time points when compared to VRP-034 (Fig. 3). This trend suggests a more pronounced inflammatory response in renal tubules treated with marketed PMB.

### Activation of caspases and cytochrome C

**Fig 4:**
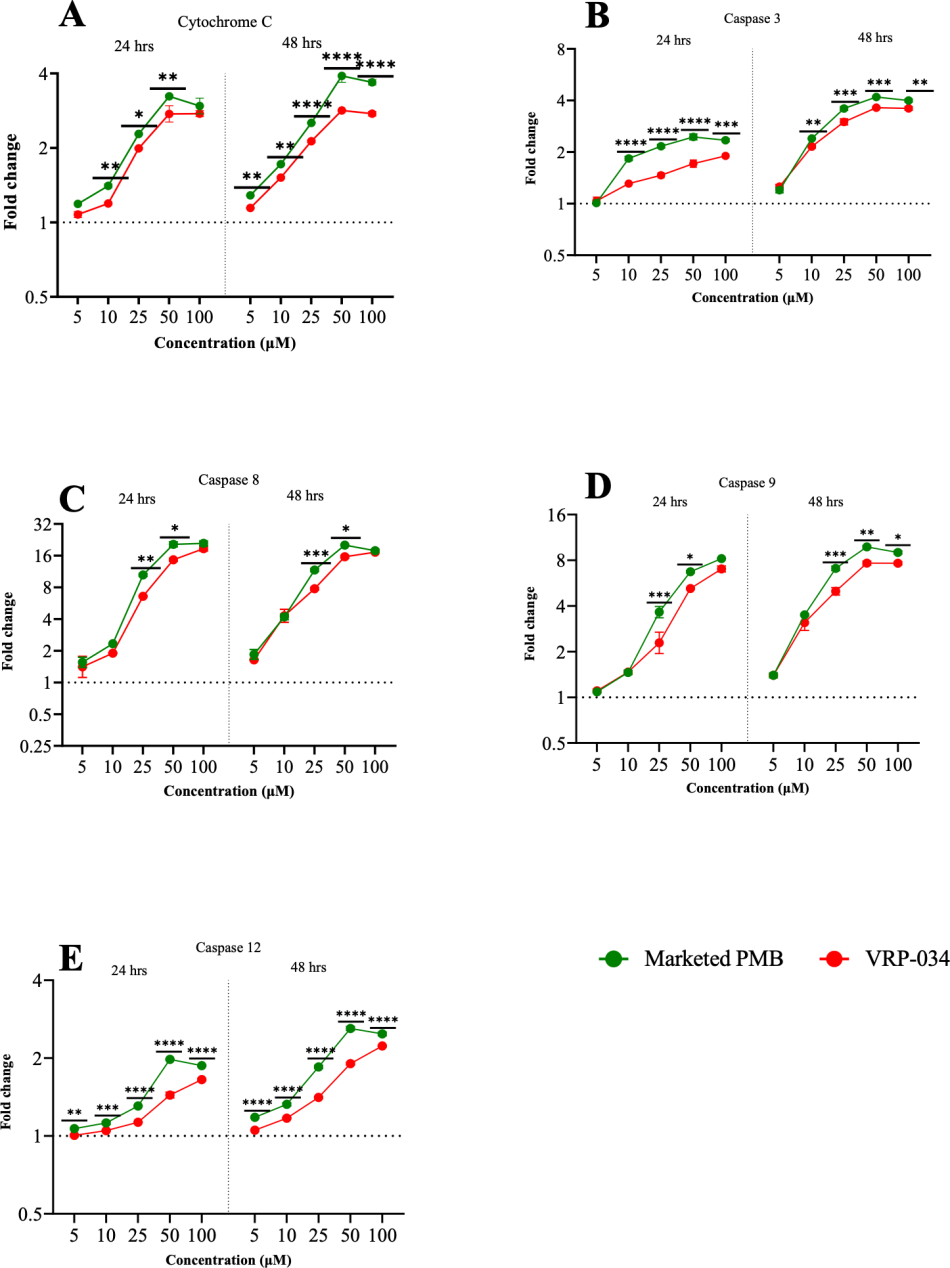
Cytochrome C and caspase levels in MPS after 24- and 48-h exposures with marketed PMB and VRP-034. (A–E) Cytochrome C and caspase levels after 24 and 48 h of exposure with different concentrations of marketed PMB and VRP-034. Data presented as the geo-mean (±SD) of the fold change from control. *P* values are calculated for the log-transformed fold-change values between the two drugs at a particular time point. Significance is denoted by *P < 0.05, **P < 0.01, ***P < 0.001, ****P < 0.0001.

Cytochrome C levels indicated a time- and dose-dependent increase within the MPS system. Marketed PMB showed significantly greater (*P* ≤ 0.05) cytochrome C starting at 10 µM compared to VRP-034 (Fig. 4). Subsequently, the activities of four regulatory caspases, caspase-3, caspase-8, caspase-9, and caspase-12, were measured to explore the complete range of caspase activation events in the cytochrome C-initiated proteolytic cascade. A concentration-dependent increase in the activity of these caspases was observed, with caspase-3 activity showing a significant rise (*P* ≤ 0.05) from 10 µM concentrations of marketed PMB compared to VRP-034 (Fig. 4). Similarly, caspase-8 and caspase-9 activities were significantly higher (*P* ≤ 0.05) from 25-µM concentrations onward. Caspase-12 activity also showed a significant increase (*P* ≤ 0.05) from concentrations of 5 µM of marketed PMB compared to VRP-034, underscoring a heightened caspase activity in cells treated with marketed PMB (*P* < 0.05).

### Morphology assessment of RPTEC/hTERT-1 cells in chip/MPS after 48-h exposure to VRP-034 and marketed PMB

**Fig 5:**
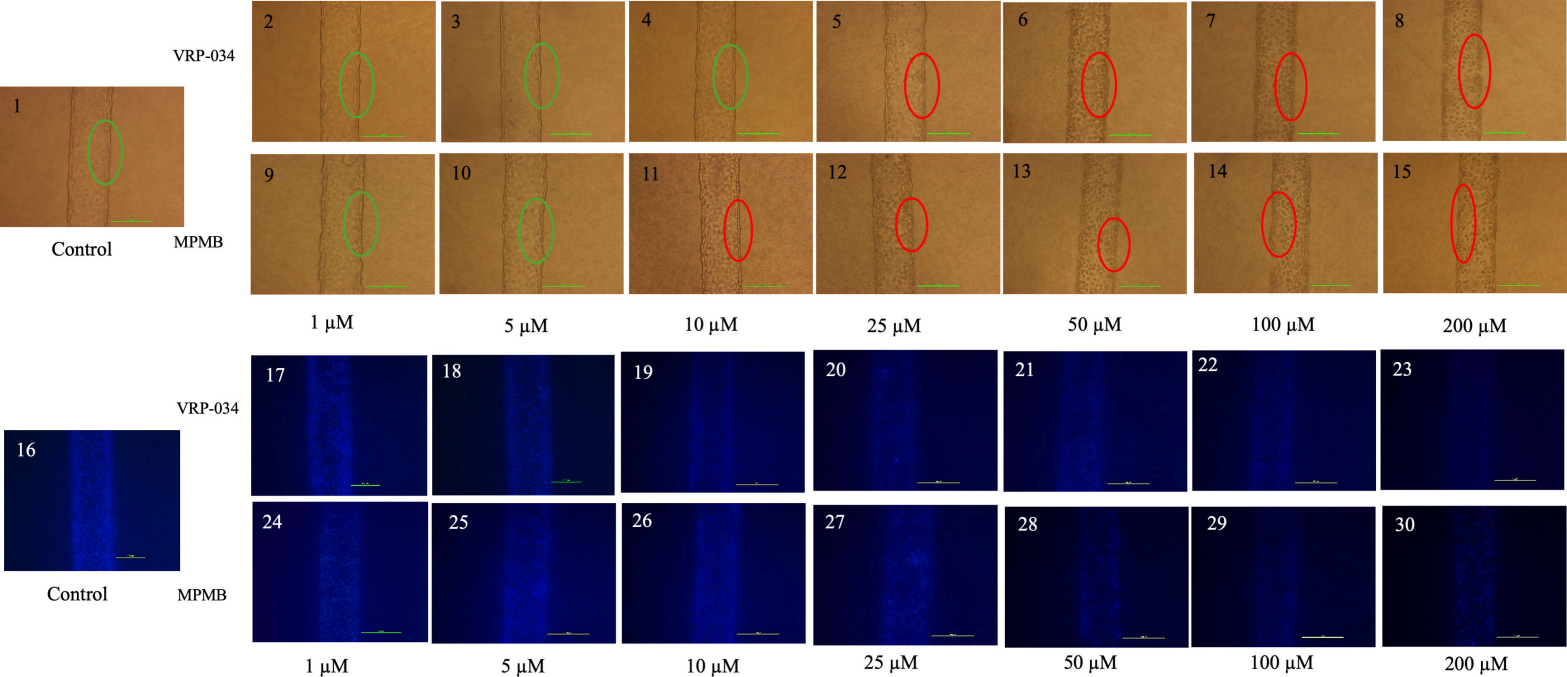
Representative image of morphology assessment of RPTEC/hTERT-1 cells after 48 h of exposure of VRP-034 and marketed PMB. Phase contrast and Hoechst-stained morphology of RPTEC/hTERT-1 cells in MPS after 48 h of exposure with VRP-034 and marketed PMB. Live morphology representative images of the tubules were taken on a Nikon microscope at scale bar: 100 µm (×20 magnification). In phase-contrast images, green circles show intact tubular integrity, and red circles denote loss of epithelial morphology and tubular integrity. Phase contrast: control (1), VRP-034 (2–8), and marketed PMB (9–15). Hoecsht-stained nuclei: Hoecsht stained to visualize nuclei by fluorescence microscopy using a 4',6-diamidino-2-phenylindole (DAPI) filter: control (16), VRP-034 (17–23), and marketed PMB (24–30). Hoecsht-stained images have been brightened for better visibility.

Control samples visualized under both phase contrast and fluorescent microscopy showed RPTEC/hTERT1 cells with well-differentiated, confluent, intact tubular epithelial morphology, and clear luminal structures within the MPS (Fig. 5, panel 1). Exposures to 1- and 5-µM concentrations of VRP-034 and marketed PMB for 48 h did not significantly affect the integrity of the tubules (Fig. 5, panels 2, 3, 9, and 10). However, at a 10-µM concentration, marketed PMB began to show evidence of compromised tight junctions, alterations in epithelial morphology, and increased cell spacing, while VRP-034 exposure maintained the integrity of the tubular structures (Fig. 5, panels 4 and 11). At 25 µM, marketed PMB exposure resulted in a noticeable loss of epithelial morphology and tubular integrity, whereas VRP-034 showed comparatively milder effects (Fig. 5, panels 5 and 12). At a concentration of 50 µM or higher, both VRP-034 and marketed PMB caused severe cell death, complete loss of tubule integrity, and cell detachment (Fig. 5, panels 6 through 8 and 13 through 15).

### Assessment of cell survival (live/dead) after 48-h exposure to VRP-034 and marketed PMB

Cell viability was assessed on-chip using live/dead staining with calcein AM (a green fluorescent dye indicating live cells) and ethidium homodimer-1 (EthD-1, a red fluorescent dye marking dead cells via nucleic acid labeling).

**Fig 6:**
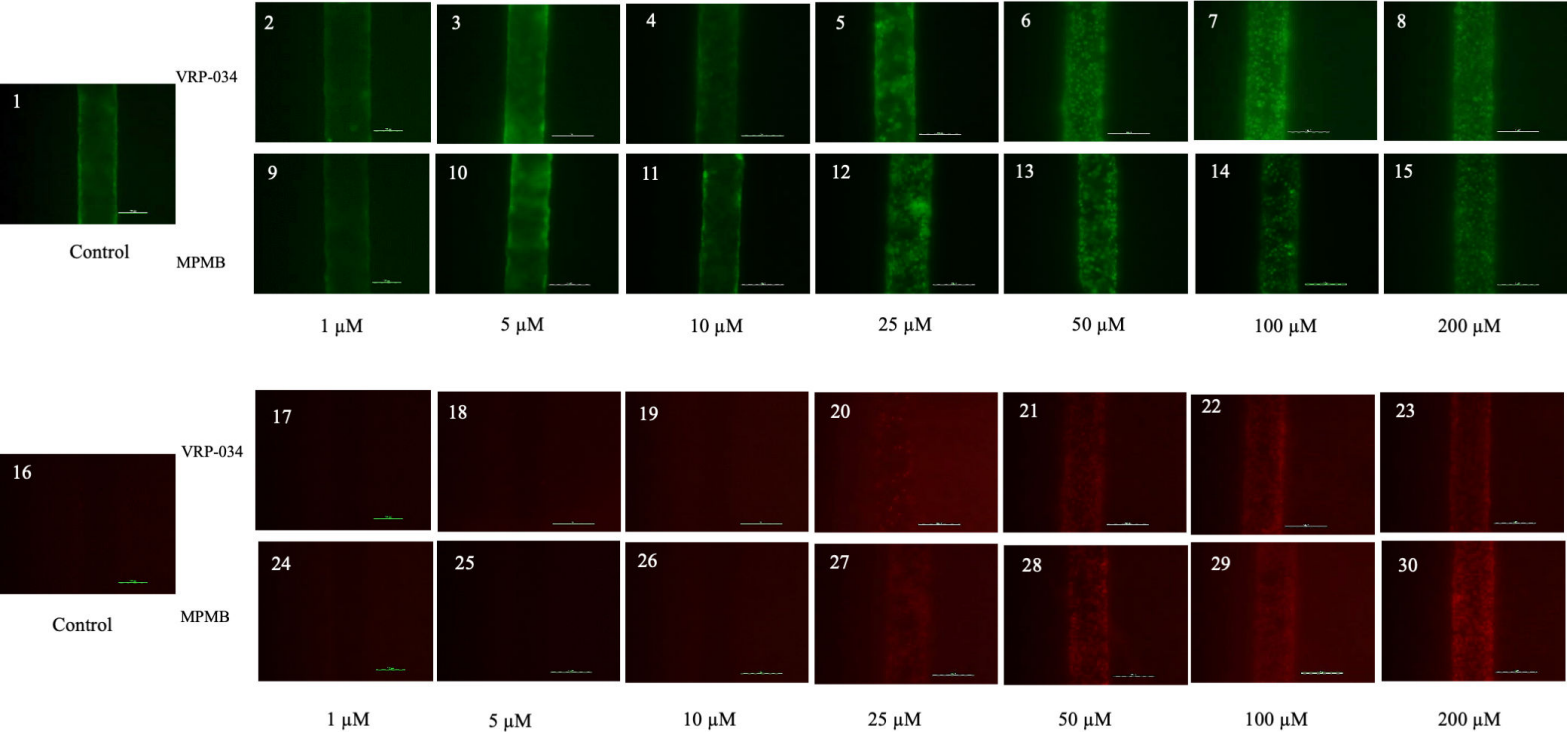
Representative image of assessment of cell survival after 48 h of exposure of VRP-034 and marketed PMB. Note: live/dead cellular staining (using ethidium homodimer and calcein AM staining) of RPTEC/hTERT-1 cells to assess observable differences in viability (green) and toxicity (red) after 48 h of exposure with VRP-034 and marketed PMB. Representative images were taken on a Nikon microscope at scale bar: 100 µm (×20 magnification). Live staining (green): control (1), VRP-034 (2–8), and marketed PMB (9–15). Dead staining (red): control (16), VRP-034 (17–23), and marketed PMB (24–30).

Up to concentrations of 10 µM for both VRP-034 and marketed PMB, the cells maintained high viability, characterized by a uniform green fluorescence (indicating live cells) and the absence of red fluorescence (indicating dead cells) (Fig. 6, panels 2 through 4 and 9 through 11). Exposure to 25 µM of marketed PMB led to significant cell death and loss of tubule integrity, while VRP-034 exposure resulted in fewer dead cells at the same concentration, as indicated by reduced red fluorescence compared to marketed PMB (Fig. 6, panels 20 and 27). Cell viability markedly decreased at concentrations of 50 µM and above for both compounds, with pronounced cell death and loss of tubule integrity, indicated by increased red fluorescence of cells (Fig. 6, panels 21 through 23 and 28 through 30).

## DISCUSSION

PMB is a crucial last-line antibiotic for treating severe infections. However, due to its dose-limiting nephrotoxicity, its use is once again on the decline (9). VRP-034, a novel PMB formulation, was devised to diminish the kidney damage linked with PMB. The objective was to assess the reduction in nephrotoxicity associated with PMB through the use of VRP-034, applying a 3D kidney MPS featuring proximal tubules composed of hTERT immortalized RPTEC-TERT-1 cells. These cells not only mirror the functional attributes of proximal tubule cells in the human kidney, including the formation of tight junctions and the expression of critical proteins and transporters necessary for reabsorption and secretion processes, but also exhibit an accurate injury response by releasing various biomarkers. Additionally, they address the limitations faced by primary RPTECs, such as replicative senescence, enhancing their utility in nephrotoxicity testing (28–32).

In recent years, the push by regulatory bodies for the adoption of novel kidney injury biomarkers such as KIM-1, cystatin-C, clusterin, NAG, NGAL, and OPN in drug safety evaluations has underscored their value as reliable indicators of drug-associated renal toxicity. These biomarkers are relevant for kidney damage due to their specific expression in response to renal injury, particularly affecting proximal tubular cells. They offer early detection capabilities beyond traditional markers like serum creatinine, aiding in assessing kidney health and drug safety (31, 33–36).

Our research utilized the MPS to gauge kidney damage by measuring these biomarkers in the effluent from the kidney chip. The outcomes revealed an upsurge in these biomarkers following exposure to marketed PMB and VRP-034 at clinically relevant doses of 10 µM (equivalent to ~13.85 µg/mL) and beyond. Notably, VRP-034 was associated with significantly lesser increases in biomarker levels compared to marketed PMB, particularly at a 25-µM concentration, where all six biomarkers significantly surged (*P* < 0.001) in a dose-dependent manner across all time points (12, 24, and 48 h) (Fig. 2). This indicates that VRP-034 may exhibit a higher *in vitro* tolerance at similar or elevated concentrations compared to PMB, possibly circumventing the narrow therapeutic margin associated with PMB. This observation is aligned with other studies where increased biomarker levels were recorded in kidney MPS following exposure to nephrotoxic agents, including polymyxins (30, 37).

Furthermore, a recent *in vitro* human model, aProximate, utilizing freshly isolated primary human proximal tubular cells (PTCs), also demonstrated a significantly higher increase in biomarkers like KIM-1, NGAL, and clusterin after treatment with marketed PMB versus VRP-034 at concentrations of 10 µM and above (*P* < 0.001) (38). It can be posited that the enhanced tolerance for VRP-034 observed *in vitro* might translate into a broader therapeutic window *in vivo*, potentially allowing for the safe administration of full recommended dosages, a prospect currently limited by existing PMB formulations.

Recent data from *in vivo* studies in Sprague-Dawley rats have shown that VRP-034 minimally affects (*P* > 0.05) biomarkers such as KIM-1, cystatin-C, urea, and creatinine, unlike marketed PMB, which significantly elevated these markers from baseline (*P* < 0.0001) (39). Moreover, a comparative analysis of the six kidney injury biomarkers outlined above showed over a 50% reduction in toxicity in animal models with VRP-034 compared to the marketed PMB formulation (40).

Additionally, animal efficacy studies and pharmacokinetic evaluations for VRP-034 yielded comparable outcomes to marketed PMB, indicating that VRP-034 does not alter the known efficacy or PK profile of PMB (41–43). No significant difference was observed in the *in vitro* efficacy of VRP-034 when compared to marketed PMB against a range of serious Gram-negative clinical isolates, including both serine carbapenemases and metallo-beta-lactamase-producing pathogens (39, 44, 45).

ROS and oxidative stress are pivotal contributors to AKI and chronic kidney disease, with mitochondria being the source of approximately 90% of cellular ROS production (46–48). Oxidative stress, marked by elevated ROS levels, is a prevalent feature across numerous renal pathologies (46, 49). Our investigation revealed an upsurge in ROS within both mitochondrial and cytosolic compartments following cell exposure to marketed PMB and VRP-034, with markedly higher mitochondrial ROS generation observed in the marketed PMB group. This underscores the pivotal role of mitochondrial ROS in PMB-induced nephrotoxicity, aligning with earlier findings that highlighted an increase in ROS production post-PMB treatment (37, 50).

Inflammatory biomarkers IL-6 and IL-8 participate in the immune response, and elevated levels suggest inflammation within the kidney tissue. These biomarkers have been correlated with AKI in clinical scenarios (51). Our data demonstrated a significant uptick in these inflammatory markers following exposure to both marketed PMB and VRP-034 at a concentration of 5 µM and above, with a notably higher increase in the marketed PMB group. This finding lends support to the notion that inflammation may precede or occur concurrently with renal tubule damage, echoing the sentiments of previous studies. The release of inflammatory biomarkers from injured proximal tubular cells into the tubular lumen has been posited as an early diagnostic indicator of AKI (52–56).

Cell health biomarkers such as ATP and LDH serve as indicators of cellular energy metabolism and cell membrane integrity, reflecting alterations in cellular function and viability. Our study further observed that elevated ROS levels correlated with increased cell membrane damage, as evidenced by heightened LDH release in the marketed PMB group compared to VRP-034, suggesting more extensive cell membrane damage (50, 57). Moreover, PMB exposure has been associated with concentration-dependent mitochondrial membrane potential loss, morphological alterations, and enhanced ROS generation (13, 16, 17). Such PMB accumulation precipitated ROS-induced apoptosis in various RPTEC cell lines (13, 15, 58). Our microscopy analysis corroborated these findings, showing dose-dependent morphological changes and apoptosis with lesser effects observed for VRP-034.

PMB-induced apoptosis engages multiple pathways, including mitochondrial (involving cytochrome C, caspase-9, and caspase-3), death receptor (Fas, Fas-L, and caspase-8), and endoplasmic reticulum pathways (caspase-12) (13–17, 58). Our findings indicated that marketed PMB elicited more pronounced activation of these apoptotic pathways compared to VRP-034. The mitochondrial dysfunction observed, evidenced by reduced ATP production, confirms the critical role of mitochondrial pathways in drug-induced nephrotoxicity, consistent with prior research (14). Furthermore, our analysis highlighted that VRP-034 significantly mitigates the activation of cytochrome C, caspase-3, caspase-8, caspase-9, and caspase-12 pathways, reducing mitochondrial dysfunction and the subsequent activation of apoptosis-triggering pathways.

This investigation suggests that VRP-034 ameliorates oxidative stress associated with PMB and bolsters critical microcirculatory functions vital for kidney health, thereby reducing PMB-linked nephrotoxicity. In our previous study (data not shown), we compared the antioxidant potential of VRP-034 and marketed polymyxin B using multiple *in vitro* assays, including total equivalent antioxidant capacity, superoxide radical scavenging, hydrogen peroxide scavenging, and reducing power assays. VRP-034 demonstrated superior antioxidant activity, with antioxidant measures showing up to a 400% improvement over marketed PMB, which can be attributed to the SMC formulation enhancing cellular protection against free radicals. This is in line with previous studies, which have shown that the co-administration of antioxidants can provide a protective shield against polymyxin-induced nephrotoxicity (14, 59, 60).

Previous research has shown that PMB primarily accumulates in renal proximal tubular cells through megalin receptors, leading to nephrotoxicity (61–63). Most studies on megalin’s role in PMB uptake have been conducted *in vitro*, focusing on apical exposure where these receptors are abundant (61, 64). However, given PMB’s high plasma protein binding, it is likely that its glomerular filtration is minimal, suggesting that *in vivo* uptake might occur predominantly via tubular secretion and potentially through basolateral membrane routes, which are less influenced by megalin (65). This implies that PMB accumulation may involve a circular pathway, with the drug being reabsorbed via apical transporters following initial basolateral uptake. Studies using megalin inhibitors may inadvertently emphasize apical transport as the primary uptake pathway, potentially overlooking contributions from basolateral transporters such as OATs and OCTs.

Moreover, research indicates that even in rodent models with knocked-out megalin/cubilin receptors, PMB toxicity is only partially reduced by about 40%, suggesting the involvement of additional uptake mechanisms (62). A study by Abdelraouf et al. (66) demonstrated that apical uptake of PMB is significantly higher than basolateral uptake under conditions where PMB was exposed equally on both sides (66). This finding suggests that when both sides are exposed to the same concentration of PMB, the apical transport dominates. However, given PMB’s distribution dynamics and limited glomerular filtration, the basolateral uptake, despite being less efficient, could still be a significant pathway for renal accumulation. This complexity implies that PMB’s renal accumulation likely involves a multifaceted mechanism, necessitating further exploration of alternative pathways beyond megalin-mediated uptake.

Our working hypothesis suggests that the mechanism of action of VRP-034 involves both the reduction of oxidative stress and the potential inhibition of membrane proteins due to the co-localization of L-arginine and PMB at the receptor site. Specifically, the SMC complex in VRP-034 may competitively bind to these receptors, potentially disrupting PMB’s binding to megalin/cubilin receptors and altering its renal uptake profile. However, this hypothesis has not yet been experimentally validated due to the unavailability of purified megalin/cubilin proteins. To address this, we are currently developing an LRP2-knockout RPTEC cell line to specifically study the role of megalin in PMB uptake, which we anticipate will provide crucial insights into PMB accumulation in proximal tubular cells. Additionally, ongoing investigations are examining PMB’s basolateral uptake in an RPTEC cell line, which is expected to exhibit more accurate transporter expression compared to the pig cell line used in previous studies (66).

The comprehensive analysis conducted within this study indicates that VRP-034 exhibits a markedly lower propensity to trigger nephrotoxicity associated with PMB in RPTEC/TERT-1 cells, as evaluated in a microphysiological system. This assertion is substantiated by the observed reductions in kidney injury biomarkers, alongside diminished oxidative stress, inflammation, and apoptotic responses when compared to cells exposed to marketed PMB. These findings underscore the potential of VRP-034 for further investigation and development as a safer therapeutic alternative for treating infections caused by Gram-negative bacteria. Such an advancement holds promise in meeting the clinical need for polymyxin antibiotics that mitigate the risk of nephrotoxicity, potentially facilitating the use of full therapeutic dosages of PMB in human patients with an improved safety profile.

## MATERIALS AND METHODS

### Drugs, media, kits, and chemicals

The study utilized a range of materials, including VRP-034 (batch no: VMRC/RD/22F009, manufacturing date [MFG]: June 2022, expiry date [EXP]: May 2024; Venus Medicine Research Centre, Himachal Pradesh, India) and marketed PMB (batch no: 2421014, MFG: November 2021, EXP: October 2024; Bharat Serum and Vaccines, Mumbai, India). Both drugs were prepared as stock solutions in distilled water and further diluted in cell culture media. Additionally, various supplies were sourced: Dulbecco's Modified Eagle's Medium (DMEM):F12 medium (ATCC 30-2006), hTERT RPTEC growth kit (ATCC ACS-4007), trypsin-EDTA for primary cells (ATCC PCS-999-003), and soybean trypsin inhibitor (ATCC 30–2104) from ATCC, USA.

Kits for specific assays like KIM-1 (ab235081), cystatin C (ab119589), clusterin (ab174447), LDH (ab102526), annexin V-FITC/apoptosis staining detection kit (Ab-14085), and ATP (ab83355) were obtained from Abcam, Cambridge, MA, USA. Osteopontin (DOST00), NGAL (DLCN20), IL-6 (D6050), and IL-8 (D8000C) kits were obtained from R&D Systems (Minneapolis, MN, USA). The NAG (MBS725099S) kit was obtained from My Biosource Inc. (SanDiego, USA). CellRox Green Reagent (C10444), MitoSOS Red mitochondrial superoxide indicator (M36008), LIVE/DEAD Viability/Cytotoxicity Kit for mammalian cells (MP 03224), geniticin, and Hoechst 33342 stain were obtained from Life Technologies Corporation, USA. Human caspase-8 (BMS2024), caspase-9 (BMS2025), caspase-3 (KHO1091), and cytochrome human ELISA kit (BMS263) were obtained from Invitrogen (Bender Medsystem Gmbh, Vienna, Austria). Caspase-12 assay kit (ab65664/Fluorometric) and caspase-3 (KM300) were purchased from Abcam and R&D Systems, respectively. Rat tail collagen I (high concentration; Corning, New York, USA; #354249) was purchased from Discovery Labware Inc., Massachusetts, USA. Collagen IV (# C5533), genipin (G4796), and fluorescein isothiocyanate-dextran 150S (1003171298) were obtained from Sigma-Aldrich, St. Louis, MO, USA. Other chemicals, unless specified, were procured from Hi-Media, Mumbai, India.

### Cell culture and cell seeding in the MPS

RPTEC/hTERT1 (ATCC-CRL-4031) cells were obtained from Manassas, USA; cultured in DMEM:F12 medium. When cells reached 80%–90% confluency, they were harvested and adjusted to a concentration of 10^4^ cell/µL. The TSC chips and microfluidic system were acquired from Nortis Bio. For cell seeding, collagen matrices were prepared and injected into the channels of the TSC chips for cell adhesion. After an overnight attachment, channels were perfused with media to wash out non-adherent cells, and the perfusion rate was adjusted for tubule formation over 7–9 days. Tubule development was monitored daily, and channels with high viability and confluence were selected for drug treatment.

### Drug exposure or dosimetry to cells cultured in TSC or MPS

The drug exposur or dosimetry,involved in this study commenced on day 0, 9 days following the seeding of RPTEC/hTERT1 cells within the MPS system. Both VRP-034 and marketed PMB were exposed to the cells across seven different concentrations [1 µM (equivalent to ~1.38 µg/mL), 5 µM (~6.92 µg/mL), 10 µM (~13.85 µg/mL), 25 µM (~34.62 µg/mL), 50 µM (~69.25 µg/mL), 100 µM (~138.5 µg/mL), and 200 µM (~277 µg/mL)] over a period of 48 h within the MPS framework. It is important to note that the concentrations for VRP-034 were exclusively based on PMB-equivalent concentrations. Specifically, 1 µM of VRP-034 refers to 1 µM of the sulfate salt of PMB, making it equivalent to 1 µM of marketed PMB. Separately, concentrations ranging from 1 to 25 µM were selected based on reported Cmax values of standard PMB at clinically administered doses (0.75–1.5 mg/kg), typically resulting in concentrations of 2–14 µg/mL (61, 67, 68). Higher concentrations (>25 µM) were included to cover a broader concentration spectrum. Following administration, kidney injury, inflammatory, and cell health biomarkers were systematically analyzed at intervals of 12, 24, and 48 h from the cell culture effluent. Moreover, cell staining to identify apoptosis/necrosis and the quantification of ROS were conducted after the 48-h drug exposure period. In addition, analyses using the supernatant/cell extract were performed for caspase assays (targeting caspase-3, caspase-8, caspase-9, and caspase-12) and for the detection of cytochrome C within the MPS system containing RPTEC/hTERT1 cells.

### Biomarker analysis

The assessment of all cell health (ATP and LDH), kidney injury (KIM-1, cystatin-C, clusterin, NAG, NGAL, and OPN), and inflammatory (IL-6 and IL-8) biomarkers was conducted on effluents from kidney MPS/chips following exposure to varying concentrations of marketed PMB, VRP-034, and a control at 12, 24, and 48 h using commercially available kits. Analyses were performed in either duplicate or triplicate to ensure accuracy.

For the ATP assay, 50 µL of effluent (either undiluted or diluted ranging from 1:2 to 1:8) was combined with the ATP assay master mix and incubated at room temperature away from light for 30 min. Absorbance was measured at 570 nm using a microplate reader (Tecan, Japan). The concentration of ATP was deduced by interpolating from a standard curve and adjusted for dilution factor, then expressed in nanomole per milliliter.

For the LDH assay, 50 µL of effluent (undiluted or diluted from 1:2 to 1:8) was mixed with an equal volume of LDH reaction mixture and incubated at room temperature for 30 min. The reaction was then halted by adding 50 µL of stop solution, and absorbance at 450 nm was measured using a microplate reader (Tecan). Corrected absorbance values were calculated by subtracting the mean blank absorbance from all standard and sample readings. These values were used to construct a standard curve by plotting corrected absorbance (*y*-axis) against the NADH standard concentration (*x*-axis) to determine LDH activity.

Activity of LDH in the test samples was calculated using the formula LDH activity (nmol/min/mL) = (*B*Δ*T* × *V*) × *D*; where *B* is the amount of NADH in the sample well calculated from the standard curve (nmol); Δ*T* is the reaction time (minute); *V* is the original sample volume added into the reaction well (mL); and *D* is the dilution factor.

Kidney injury and inflammatory biomarkers were quantified in the effluent according to the manufacturer’s protocols. Dilutions of 1:2 were made for KIM-1, clusterin, and cystatin C assays, while neat effluent was used for NAG, NGAL, and OPN assays. NAG levels were measured in duplicate, whereas other biomarkers were analyzed in triplicate. Absorbance values were adjusted by subtracting average blank absorbance, and a standard curve was plotted to fit a four-parameter logistic (4-PL) curve using GraphPad Prism (v.9.0; GraphPad Software, San Diego, CA, USA). Concentrations of biomarkers were interpolated from these curves, adjusted for any necessary dilution, and reported in picogram per milliliter, with the exception of OPN, which was reported in nanogram per milliter.

Measurements for all biomarkers were taken at three distinct time points—12, 24, and 48 h—following exposure to VRP-034, marketed PMB, and the control group.

### Caspase and cytochrome C assay

The activities of caspases (caspase-3,–8, −9, and −12) and cytochrome C were quantified in the cell culture supernatant or cell extract using specific assay kits. RPTEC/hTERT1 cells, cultivated in 24/96-well plates, were subjected to a range of concentrations of marketed PMB and VRP-034 (0, 1, 5, 10, 25, 50, 100, and 200 µM). Within the MPS, cells were exposed to 0, 5, 10, 25, 50, and 100 µM concentrations of both PMB and VRP-034. The detection of absorbance/fluorescence was carried out using a multimode microplate reader (Tecan). To ensure accuracy, the average blank absorbance was subtracted from the absorbance values of standards, controls, and samples. A standard curve was established by plotting the mean absorbance against concentration, which was then fitted with a 4-PL curve via GraphPad Prism (v.9.0), applied for caspase-3, caspase-8, caspase-9, and cytochrome C analyses. The concentrations of caspase and cytochrome C were determined by interpolation from the standard curve, with adjustments made for any dilution. In the case of caspase-12, fluorescence intensity rather than concentration was used for statistical analysis. Concentrations of caspase and cytochrome C were reported in picogram per milliliter, with the exception of caspase-12, where fluorescence intensity was reported.

### Assessment of morphology of RPTEC/hTERT-1 and cell survival using live/dead staining

The LIVE/DEAD Viability/Cytotoxicity Kit was employed to distinguish between viable and dead cells, adhering to the manufacturer’s protocol. The staining medium, composed of calcein AM (2 µM), EthD-1 (4 µM), and Hoechst 33342 (1 μg/ml) was perfused through TSCs at a flow rate of 1 µL/min for 90 min. This procedure was conducted in an incubator set to a humidified atmosphere of 37°C and 5% CO_2_. Following the perfusion, the flow was halted, allowing the chips to rest undisturbed for an additional 30 min. After this period, fluorescent microscopy images were captured using a Nikon Eclipse Ti-S to visualize the cells. Live cells were indicated by green fluorescence; dead cells were indicated by red fluorescence; and cell nuclei were marked in blue with Hoechst 33342 staining. For each experimental condition, encompassing various concentrations and time points, three images were acquired at 20x magnification. These images maintained uniform exposure times and light intensity settings to ensure consistency. Subsequent analysis of these images was carried out using Nikon Eclipse software, specifically focusing on quantifying the intensities of green and red fluorescence to assess the viability and cytotoxicity within the cell populations.

### Statistical analysis

The data are presented as geometric means (±standard deviation) representing the fold change relative to the control group. Comparative analysis of these means was conducted utilizing two-way analysis of variance, with further post hoc testing via Sidak’s multiple comparisons test, executed within GraphPad Software. The significance of differences between the two drugs at specific time points was determined by calculating *P* values for the log-transformed fold-change values. Significance levels are indicated as follows: *P < 0.05, **P < 0.01, ***P < 0.001, ****P < 0.0001.
